# Maternal immune activation produces neonatal excitability defects in offspring hippocampal neurons from pregnant rats treated with poly I:C

**DOI:** 10.1038/srep19106

**Published:** 2016-01-08

**Authors:** Eti Patrich, Yael Piontkewitz, Asher Peretz, Ina Weiner, Bernard Attali

**Affiliations:** 1 Department of Physiology & Pharmacology, Sackler Faculty of Medicine, Tel Aviv University, Tel Aviv 69978, Israel; 2 Department of Psychology, Gordon Faculty of Social Sciences, Tel Aviv University, Tel Aviv 69978, Israel; 3 Sagol School of Neuroscience, Tel Aviv University, Tel Aviv 69978, Israel; 4 Strauss Center for computational Neuroimaging, George S. Wise Faculty of Life Sciences, Tel Aviv University, Tel Aviv 69978, Israel

## Abstract

Maternal immune activation (MIA) resulting from prenatal exposure to infectious pathogens or inflammatory stimuli is increasingly recognized to play an important etiological role in neuropsychiatric disorders with neurodevelopmental features. MIA in pregnant rodents induced by injection of the synthetic double-stranded RNA, Poly I:C, a mimic of viral infection, leads to a wide spectrum of behavioral abnormalities as well as structural and functional defects in the brain. Previous MIA studies using poly I:C prenatal treatment suggested that neurophysiological alterations occur in the hippocampus. However, these investigations used only juvenile or adult animals. We postulated that MIA-induced alterations could occur earlier at neonatal/early postnatal stages. Here we examined the neurophysiological properties of cultured pyramidal-like hippocampal neurons prepared from neonatal (P0-P2) offspring of pregnant rats injected with poly I:C. Offspring neurons from poly I:C-treated mothers exhibited significantly lower intrinsic excitability and stronger spike frequency adaptation, compared to saline. A similar lower intrinsic excitability was observed in CA1 pyramidal neurons from hippocampal slices of two weeks-old poly I:C offspring. Cultured hippocampal neurons also displayed lower frequency of spontaneous firing, higher charge transfer of IPSCs and larger amplitude of miniature IPSCs. Thus, maternal immune activation leads to strikingly early neurophysiological abnormalities in hippocampal neurons.

Epidemiology studies indicate that maternal immune activation (MIA) resulting from inflammatory stimuli, viral or bacterial infections of pregnant mothers play an important etiological role in neuropsychiatric and neurological disorders with neurodevelopmental features including schizophrenia, autism, bipolar disorder, mental retardation, and cerebral palsy^1^,^2^,^3^,^4^,^5^,^6^,^7^,^8^,^9^,^10^. However, while these associations provide some rationale to suggest that prenatal infection might contribute to the origin of these disorders, they do not prove causality^1^,^2^.

Although schizophrenia has a significant genetic component, many studies support the notion that schizophrenia is a neurodevelopmental disorder and that environmental factors may also provide substantial triggers for the disease to manifest^11^,^12^,^13^. Recently, epigenetic dysfunction in response to a variety of environmental factors has been suggested to play a major role in the etiology of schizophrenia, providing a link between the environment and the genome in the pathogenesis of the disease^14^,^15^,^16^. These combined genetic and environmental factors may alter neurodevelopmental processes that significantly precede the onset of clinical symptoms, which appear only at the late adolescence or early adulthood.

One of the most prevalent methods to mimic maternal infection in animal models is to treat pregnant females with molecules that target toll-like receptors (TLRs), leading to activation of the innate immune response^17^,^18^. Prenatal treatment with either the TLR3 agonist polyinosinic-polycytidylic acid (poly (I:C)), an analog of double stranded RNA, or with the TLR4 agonist, Gram-negative lipopolysaccharide (LPS), produces brain and behavior changes that feature some aspects of neurodevelopmental disorders such as schizophrenia and autism^19^. One of the major environmental risk factors for schizophrenia is MIA subsequent to maternal viral or bacterial infections during pregnancy^3^,^4^,^5^,^7^,^10^. Immune activation of pregnant rodents by injection of poly I:C, which mimics viral infection, leads in offspring to a broad range of schizophrenia-relevant behavioral and neuroanatomical deficits that exhibit a characteristic emergence at adulthood^20^,^21^,^22^,^23^,^24^,^25^. We previously found that exposure of pregnant rats to poly I:C leads to post-pubertal emergence of structural abnormalities, namely, enlarged lateral ventricles and smaller hippocampus in the offspring^26^,^27^,^28^. Structural and functional abnormalities of the hippocampus are a ubiquitous feature of several major adult-onsets neuropsychiatric disorders, including schizophrenia, depression and bipolar disorder, and are believed to mediate at least some of the cognitive impairments in these diseases^29^,^30^,^31^,^32^,^33^,^34^,^35^,^36^,^37^,^38^,^39^.

Several laboratories attempted to investigate in rodent models the hippocampal neurophysiological alterations following MIA. These models were based on infection of pregnant mothers at mid-gestation with either lipopolysaccharide (LPS) or polyI:C. Extracellular and intracellular recording data indicated that MIA leads in the offspring to hippocampal alterations in synaptic transmission and long-term plasticity^40^,^41^,^42^,^43^,^44^,^45^,^46^. However, these studies yielded inconsistent results and no work examined the impact of MIA on the intrinsic excitability of hippocampal pyramidal neurons. In addition, these previous investigations were performed only in juvenile or adult animals. It is therefore unknown whether neurophysiological alterations following MIA could occur earlier in neonatal/early postnatal hippocampal neurons.

To test this hypothesis and circumvent the impossibility of preparing hippocampal slices from newborn animals, we cultured hippocampal neurons from postnatal days 0–2 male offspring of saline-injected and poly I:C-treated pregnant rats. We examined whether MIA affects the intrinsic excitability and the synaptic properties of pyramidal-like hippocampal neuron. We show that compared to controls, hippocampal neurons from newborn offspring of poly I:C-injected dams exhibited lower intrinsic excitability. Similarly, a decreased intrinsic excitability was observed in CA1 pyramidal neurons from hippocampal slices of two weeks-old poly I:C offspring. Cultured hippocampal neurons displayed lower frequency of spontaneous spike discharge, larger spontaneous inhibitory post-synaptic currents (sIPSCs) and larger amplitude of miniature IPSCs (mIPSCs). Taken together, our data suggest that maternal immune activation leads to neurophysiological abnormalities in hippocampal neurons derived from neonatal offspring animals.

## Results

### Intrinsic excitability properties of hippocampal neurons cultured from neonatal offspring of polyI:C- and saline-treated pregnant rats

To test whether MIA could affect neonatal functions of offspring neurons, we compared the electrophysiological properties of cultured hippocampal neurons prepared from male offspring of poly I:C treated mothers at PND0-2 days with those from saline-injected mothers. Primary dissociated hippocampal cultures were maintained for two weeks *in vitro*, after which whole-cell patch-clamp recordings were performed on neurons with pyramidal-like morphology. This culture system has been widely used to investigate the mechanisms underlying synaptogenesis, synaptic transmission and plasticity^47^,^48^. Hippocampal neurons obtained from offspring of poly I:C- and saline-injected mothers exhibited similar resting membrane potential (Table 1).

**Figure 1:**
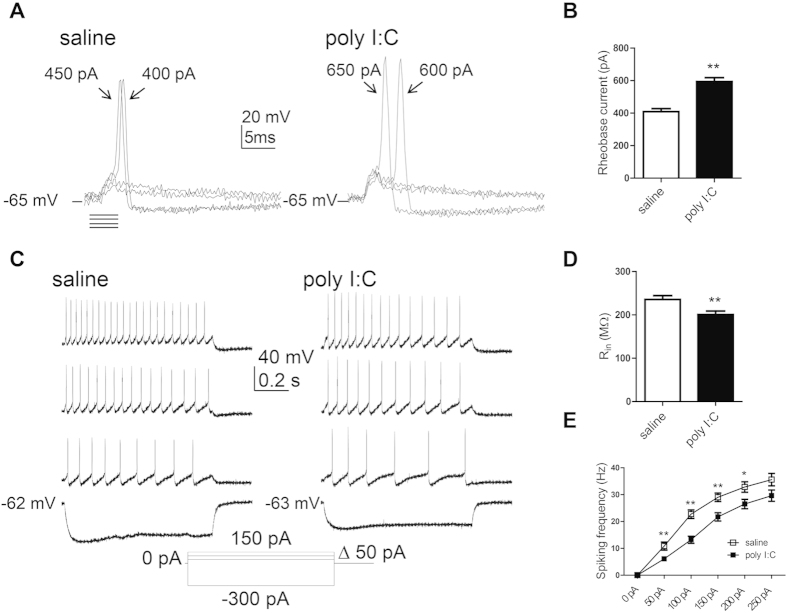
Cultured hippocampal neurons from offspring of polyI:C-injected mothers exhibit lower intrinsic excitability properties. (**A)** Representative superimposed traces of single action potentials from cultured hippocampal neurons from offspring of saline (left panel) and polyI:C (right panel) -injected mothers. Solitary spike was evoked by 2 ms squared pulse injection of current steps starting from 200 pA in 50 pA increments, in the presence of glutamate and GABA_A_ ionotropic receptors blockers (10 μM NBQX, 10 μM AP-5, 30 μM picrotoxin and 10 μM bicuculline methyl iodide). The line represents the starting resting potential before current injection. (**B**) Significantly higher current injection was required to evoke a single spike in cultured hippocampal neurons from offspring of polyI:C-injected mothers compared to those from offspring of saline-injected mothers (594 ± 25 pA, n = 54 from 6 poly I:C-injected mothers versus 409 ± 19 pA, n = 44 from 6 saline-injected mothers, t(96) = 5.69, p < 0.0001). (**C)** Representative traces of evoked spiking activity of cultured hippocampal neurons from offspring of saline (left panel) and polyI:C (right panel) -injected mothers. Spiking activity was evoked by 800 ms squared pulse injection of series of current steps starting from −300 pA up to 250 pA in 50 pA increments, in the presence of the glutamate and GABA_A_ ionotropic receptors blockers. (**D**) Cultured hippocampal neurons from offspring of polyI:C-injected mothers exhibited significantly lower membrane input resistance compared to those of saline-injected mothers (Rin = 200 ± 8 MΩ, n = 56 from 6 polyI:C-injected mothers versus Rin = 236 ± 9 MΩ, n = 41 from 6 saline-injected mothers; t(95) = −2.79, p < 0.01). (**E**) frequency-current plots showing that the firing frequency in cultured hippocampal neurons from offspring of polyI:C-injected mothers is lower compared to that from offspring of saline-injected mothers. Repeated ANOVA yielded main effects of current injection, prenatal treatment and significant interaction between current injection and prenatal treatment (F(5, 450) = 235.2, p < 0.0001; F(1, 90) = 11.81, p < 0.001 and F(5, 450) = 3.7, P < 0.003 respectively; post hoc p’s < 0.05; n = 55 from 6 polyI:C-injected mothers versus n = 37 from offspring of 6 saline-injected mothers).

**Figure 2:**
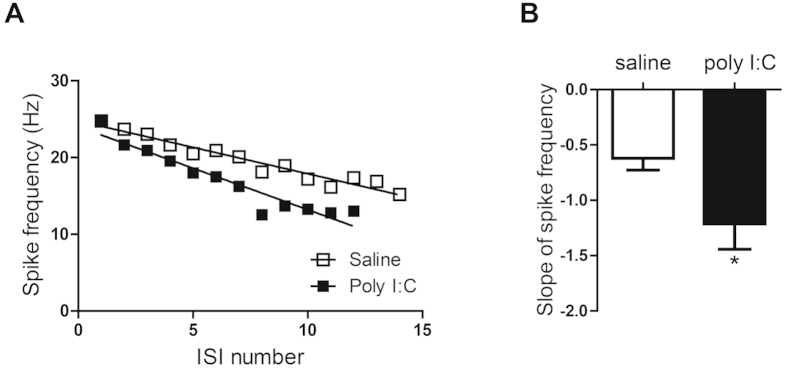
Cultured hippocampal neurons from offspring of polyI:C-injected mothers exhibit stronger spike frequency adaptation. (**A**) Representative frequency-interspike interval (ISI) plot showing the stronger spike frequency adaptation as calculated by the slope of the linear regression of the spike frequency as a function of the ISI number. (**B**) Hippocampal neurons from offspring of polyI:C-injected mothers exhibit a larger slope (S) of spike frequency adaptation than those from offspring of saline-injected moms (S = −1.21 ± 0.22, n = 35 from 6 polyI:C-injected mothers versus S = −0.62 ± 0.11, n = 38 from 6 saline-injected mothers; t(71) = −2.25, p < 0.05).

To check the intrinsic excitability of hippocampal neurons derived from offspring of both groups, we blocked spontaneous ongoing synaptic activity by using blockers of AMPA, NMDA and GABA_A_ receptors (see methods). Neurons were injected with a series of very brief incremental depolarizing current pulses to evoke a solitary spike. The minimum amount of current injection (rheobase current) necessary to trigger a single action potential was then compared between the two groups (Fig. 1A,B and Table 1). A significantly larger rheobase current was needed to evoke a solitary spike in neurons from offspring of poly I:C-injected mothers compared to that of saline-injected mothers (594 ± 25 pA, n = 54 versus 409 ± 19 pA, n = 44, t(96) = 5.69, p < 0.0001). While no difference was found in the action potential threshold, the spike amplitude was significantly higher in offspring from poly I:C-treated dams than those from saline-injected dams (87.2 ± 1.7 mV, n = 54 versus 81.2 ± 1.7 mV, n = 44, t(96) = 2.42, p < 0.02) (Fig. 1A and Table 1). Also, the action potential width at threshold was significantly shorter in offspring from poly I:C-treated-mothers (2.10 ± 0.08 ms, n = 54 versus 2.40 ± 0.15 ms, n = 44, t(96) = −2.04, p < 0.05; Table 1). Hippocampal neurons obtained from the offspring of poly I:C-treated mothers exhibited significantly lower membrane input resistance compared to that of saline-injected mothers (Fig. 1C,D and Table 1; 200.5 ± 8.5 MΩ, versus 235.6 ± 8.8 MΩ, respectively; t(95) = −2.79, p < 0.01). Results indicate a reduced firing frequency in hippocampal neurons from offspring of poly I:C-treated mothers, compared to saline, following current injection from 50 pA to 200 pA. Repeated ANOVA yielded main effects of current injection, prenatal treatment and significant interaction between current injection and prenatal treatment (Fig. 1C,E,F(5, 450)=235.2, p < 0.0001; F(1, 90)=11.81, p < 0.001 and F(5, 450) = 3.7, P < 0.003 respectively; post hoc of 50–200 pA, p’s < 0.05; n = 55 from poly I:C versus n = 37 from saline). Figures 1 and 2 show that the reduced spike discharge frequency evoked in offspring of poly I:C-injected dams was also accompanied by a significantly larger spike frequency adaptation as quantified by the slope of spike frequency (Fig. 2B; S = −1.21 ± 0.23, n = 38 versus S = −0.62 ± 0.1, n = 35; t(71) = −2.25, p < 0.05). So far, the results suggest that cultured hippocampal neurons from PND0-2 offspring of poly I:C-treated mothers exhibit lower intrinsic excitability characteristics than those from offspring of saline-treated mothers.

### Intrinsic excitability properties of CA1 pyramidal neurons from acute hippocampal slices of 2 weeks-old offspring of poly I:C- and saline-treated pregnant rats

**Figure 3:**
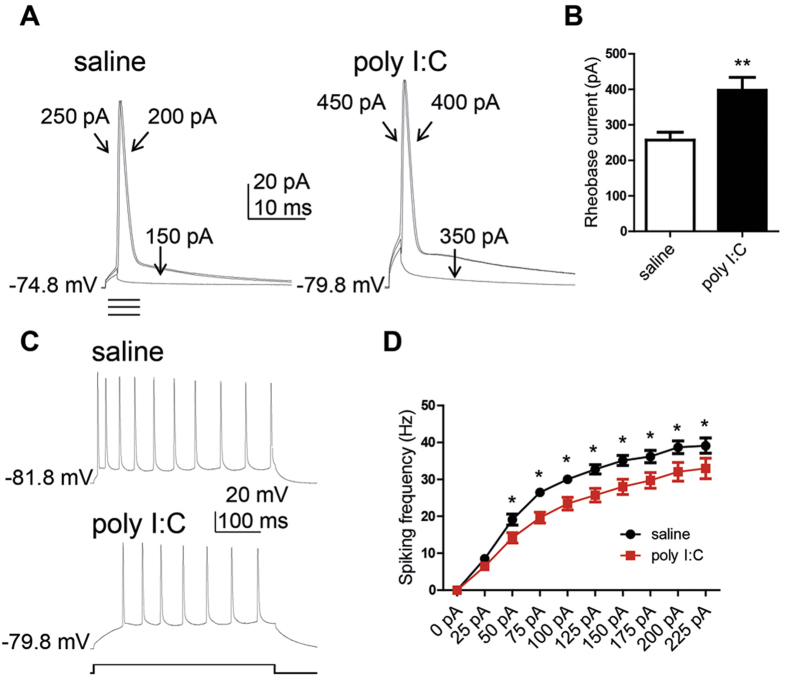
CA1 pyramidal neurons of acute hippocampal slices from juvenile offspring of polyI:C-injected mothers exhibit lower intrinsic excitability. (**A**) Representative superimposed traces of single action potentials from cultured hippocampal neurons from offspring of saline (left panel) and polyI:C (right panel) -injected mothers. Solitary spike was evoked by 2 ms squared pulse injection of current steps starting from 50 pA in 50 pA increments. (**B**) CA1 pyramidal neurons from offspring of polyI:C-injected mothers have significantly higher rheobase current compared to those from offspring of saline-injected mothers (398 ± 36 pA, n = 24 from 7 polyI:C-injected mothers versus 257 ± 22 pA, n = 27 from 6 saline-injected mothers, t(49) = 3.34, p < 0.001). (**C**) representative traces of evoked spiking activity of CA1 pyramidal neurons from offspring of saline (top)- and polyI:C (lower)-injected mothers, respectively. Spiking activity was evoked by 400 ms squared pulse injection of series of current steps starting from 0 pA up to 225 pA in 25 pA increments. (**D**) The spike frequency of offspring from polyI:C-injected mothers is lower compared to that from saline-injected mothers. Repeated ANOVA yielded main effects of stimulation current, prenatal treatment and significant interaction between stimulation current and prenatal treatment (F(9, 369) = 272.8, p < 0.0001; F(1, 41) = 10.86, p < 0.005; F(9, 369) = 2.65, p < 0.006, respectively; post hoc p’s < 0.05; n = 21 from offspring of 6 polyI:C injected mothers versus, n = 22 from offspring of 6 saline injected mothers).

Though patch-clamp recordings were performed on neurons with pyramidal-like morphology, we sought to determine whether similar excitability changes could be observed in early postnatal CA1 pyramidal neurons from acute slices where the integrity of the hippocampal architecture is largely maintained. Two weeks-old male offspring (PND14-16) from poly I:C and saline-injected mothers were sacrificed and acute hippocampal slices were prepared. Like in dissociated hippocampal neurons, a significantly larger rheobase current was needed to evoke a solitary spike in CA1 pyramidal neurons of offspring slices from poly I:C-injected mothers compared to that of saline-injected dams (Fig. 3A,B; 398 ± 36 pA, n = 24 versus 257 ± 22 pA, n = 27, t(49) = 3.34, p < 0.001). The frequency of evoked spike discharge in response to sustained depolarizing current injection was lower in CA1 pyramidal neurons of offspring from poly I:C treated mothers. Repeated ANOVA yielded main effects of current injection, prenatal treatment and significant interaction between current injection and prenatal treatment (Fig. 3C,D; F(9, 369) = 272.8, p < 0.0001; F(1, 41) = 10.86, p < 0.005; F(9, 369) = 2.65, p < 0.006, respectively; post hoc of 50–225 pA, p’s < 0.05; n = 21 from offspring of poly I:C versus, n = 22 from offspring of saline).

### Spontaneous excitatory and inhibitory post-synaptic currents in hippocampal neurons cultured from neonatal offspring of polyI:C- and saline-treated pregnant rats

**Figure 4:**
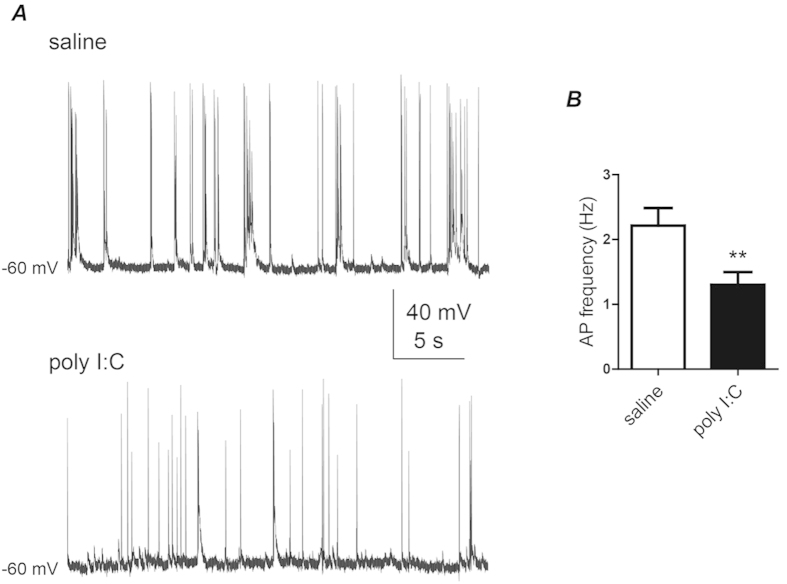
Cultured hippocampal neurons from offspring of polyI:C injected mothers exhibit lower frequency of spontaneous spike discharge. (**A**) Representative current-clamp recording of ongoing spiking activity of cultured hippocampal neurons from offspring of saline (top) and poly I:C (bottom) -injected mothers. (**B**) Cultured hippocampal neurons of offspring from polyI:C-injected mothers display significantly lower frequency of spontaneous spike discharge compared to that of offspring from saline-injected mothers (F = 1.30 ± 0.20, n = 57 from 6 polyI:C-injected mothers versus F = 2.21 ± 0.27, n = 46 from 6 saline-injected mothers; t(101) = −2.77, p < 0.01).

**Figure 5:**
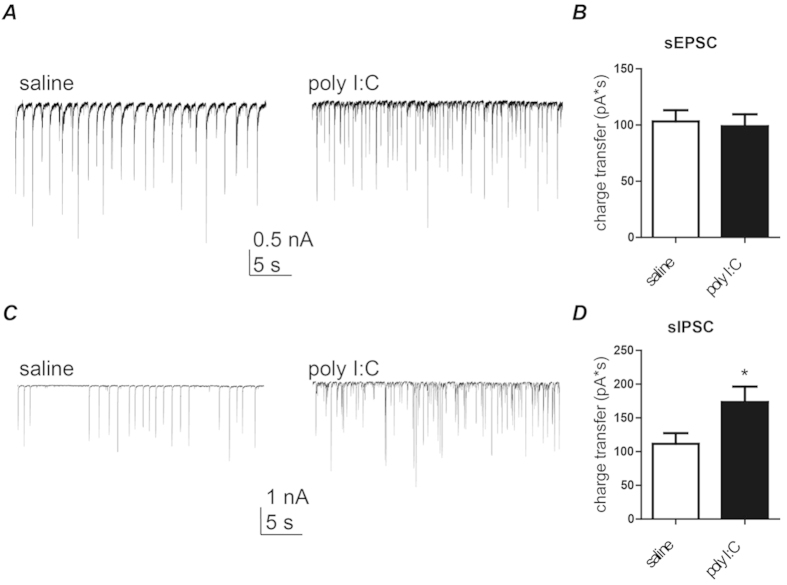
Cultured hippocampal neurons from offspring of polyI:C-treated mothers exhibit no change in sEPSC charge transfer but larger charge transfer of sIPSC. (**A**) Representative voltage-clamp recording of sEPSC of cultured hippocampal neurons from offspring of saline (left) and polyI:C (right)-injected mothers. (**B**) Cultured hippocampal neurons from the offspring of polyI:C treated mothers display similar sEPSC charge transfer compared to that of offspring from saline-treated mothers (Q = 99 ± 10 pA*s, n = 44 from 6 polyI:C-injected mothers Q = 103 ± 10 pA*s, n = 41 from 6 saline-injected mothers). (**C**) Representative voltage-clamp recording of sIPSC of cultured hippocampal neurons from offspring of saline (left) and polyI:C (right)-injected mothers. (**D**) Cultured hippocampal neurons from offspring of polyI:C-treated mothers exhibit larger sIPSC charge transfer compared to those from offspring of saline-treated mothers (Q = 173 ± 23 pA*s, n = 38 from 6 polyI:C-injected mothers versus Q = 112 ± 16 pA*s, n = 47 from 6 saline-injected mothers; t(83) = 2.25, p < 0.03).

Cultured hippocampal neurons obtained from offspring of poly I:C-injected mothers displayed a significantly lower frequency of spontaneous spike discharge than those from offspring of saline-injected mothers (Fig. 4; 1.3 ± 0.2 Hz, n = 57 versus 2.2 ± 0.3 Hz, n = 46, t(101) = −2.77, p < 0.01). Using the voltage-clamp configuration of the whole-cell patch-clamp technique, we recorded the spontaneous excitatory and inhibitory post-synaptic currents (sEPSCs and sIPSCs), which reflect the network-driven synaptic release of glutamate and GABA, respectively. Figure 5A,B shows that no quantitative difference was found in sEPSCs average charge transfer between the two groups. However, for small size sEPSCs (100–500 pA) a significantly higher frequency and lower amplitude was found in the poly I:C group as compared to saline (event frequency = 12.0 ± 2.0 Hz versus 6.9 ± 1.4 Hz, t(78) = 2.07, p < 0.05; mean amplitude = 171 ± 8 pA versus 237 ± 14 pA, t(78) = −4.3, p < 0.0001; n = 43 and n = 37, respectively). In contrast, sIPSCs from offspring of poly I:C-treated mothers displayed higher charge transfer compared to those from offspring of saline-treated mothers (Fig. 5C,D; 173 ± 23 pA*s; n = 38 versus 112 ± 16 pA*s; n = 47, t(83) = 2.25, p < 0.03).

**Figure 6:**
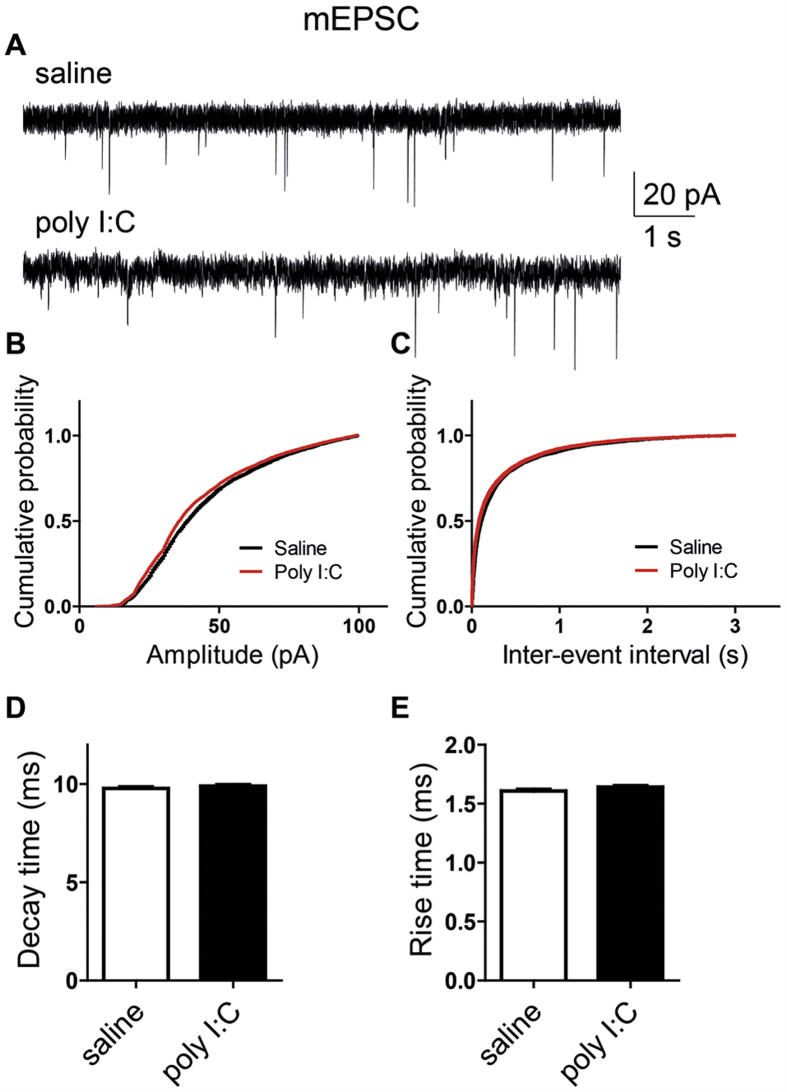
Cultured hippocampal neurons from offspring of saline and polyI:C-treated mothers exhibit similar mEPSC properties. (**A**) Representative voltage-clamp recording of mEPSCs in cultured hippocampal neurons from offspring of saline (top) and polyI:C (bottom)-injected mothers. (**B**) Cumulative probability-amplitude plots show no differences in the mEPSC amplitude of cultured hippocampal neurons from offspring of saline and polyI:C-injected mothers (n = 35 cells and n = 40 cells, respectively, in each group from 6 different mothers). (**C)** Cumulative probability-interevent interval plots show no differences in the mEPSC frequency of cultured hippocampal neurons from offspring of saline and polyI:C-injected mothers (n = 32 cells and n = 37 cells, respectively, in each group from 6 different mothers). (**D**) No differences were found in the mEPSC decay time in cultured hippocampal neurons from offspring of saline and polyI:C-injected mothers (n = 35 cells and n = 40 cells, respectively, in each group from 6 different mothers). (**E**) No differences were found in the mEPSC rise time in cultured hippocampal neurons from offspring of saline and polyI:C-injected mothers (n = 35 cells and n = 40 cells, respectively, in each group from 6 different mothers). For each recorded neuron, 200 events were analyzed.

**Figure 7:**
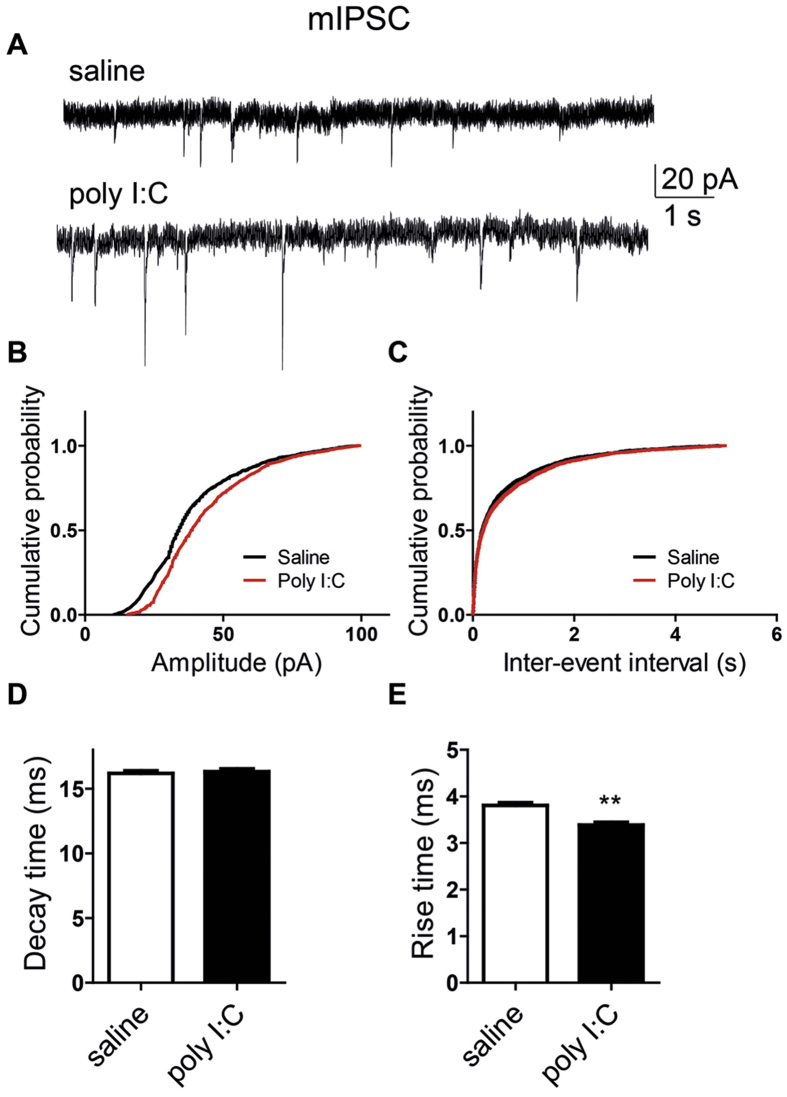
Cultured hippocampal neurons from offspring of polyI:C injected mothers exhibit larger amplitude and shorter rise time of mIPSCs. (**A**) Representative voltage-clamp recording of mIPSCs in cultured hippocampal neurons from offspring of saline (top) and polyI:C (bottom)-injected mothers. (**B**) Cumulative probability-amplitude plots show larger amplitude of mIPSCs in cultured hippocampal neurons from offspring and polyI:C-injected mothers compared to that from offspring of saline-treated moms (I = 43.4 ± 0.5 pA, n = 28 cells from 6 polyI:C-injected mothers versus I = 38.6 ± 0.5 pA, n = 30 cells from 6 saline-injected mothers; p < 0.0001). (**C**) Cumulative probability-interevent interval plots show no differences in the mIPSC frequency of cultured hippocampal neurons from offspring of saline and polyI:C-injected mothers (n = 27 cells and n = 24 cells, respectively, in each group from 6 different mothers). (**D**) no differences were found in the mIPSC decay time in cultured hippocampal neurons from offspring of saline and polyI:C-injected mothers (n = 30 cells and n = 28 cells, respectively, in each group from 6 different mothers). (**E**) Cultured hippocampal neurons from offspring of polyI:C injected mothers exhibit shorter mean rise time of mIPSCs (3.4 ± 0.06 ms, n = 28 cells from 6 polyI:C-injected mothers versus 3.8 ± 0.06 ms ,n = 30 cells from 6 saline-injected mothers; t(2733) = −4.88, p < 0.0001). For each recorded neuron, 50 events were analyzed.

We examined further a possible impact of MIA on synaptic transmission by analyzing its effects on miniature EPSCs and IPSCs (mEPSCs and mIPSCs), which reflect the spontaneous release of transmitter quanta independent of spike discharge. To that end, the cultured hippocampal neurons were bathed in solutions containing 1 μM tetrodotoxin and were held at −70 mV. Figure 6 shows that MIA did not significantly affect the amplitude, the kinetics and the frequency of mEPSCs. No difference were found in the frequency and the mean decay time of mIPSCs between the two groups (Fig. 7A,C,D). In contrast, significantly larger amplitude and shorter rise time were observed in mIPSCs from offspring of poly I:C-injected dams than those from offspring of saline-injected dams (Fig. 7A,B,E; mean amplitude = 43 ± 1 pA versus 38 ± 1 pA and mean rise time = 3.38 ± 0.06 ms versus 3.80 ± 0.06 ms, t(2733) = −4.88; n = 30 and n = 28, respectively; p < 0.0001).

## Discussion

The main findings of this study indicate that in the poly I:C model of MIA, extremely early functional abnormalities occur in hippocampal neurons cultured from neonatal offspring of poly I:C-treated moms compared to those derived from offspring of saline-treated mothers. To the best of our knowledge, our observations represent the earliest neurophysiological alterations occurring after MIA reported in the literature and they include: (1) lower intrinsic excitability and stronger spike frequency adaptation; (2) lower frequency of spontaneous spike discharge; and (3) stronger inhibitory GABAergic drive.

The lower intrinsic excitability of hippocampal neurons cultured from neonatal offspring of poly I:C-treated mothers, which is reflected by the higher rheobase current and the concomitant smaller input resistance, may involve plastic changes in voltage-gated ion channels, including Na^+^ and K^+^ channels. We observed a similar lower intrinsic excitability in CA1 pyramidal neurons from acute hippocampal slices of two weeks-old male offspring of the poly I:C group, which confirms the generality of the neonatal/early postnatal alterations in a non-dissociated hippocampal network. The reduced frequency of evoked spike discharge may be caused by an enhanced voltage-dependent inactivating K^+^ current, I_A_ or by an increased sub-threshold, non-inactivating M-type K^+^ current that plays an important role in controlling excitability of central and peripheral neurons^49^,^50^,^51^,^52^. In line with the latter assumption, stronger spike frequency adaptation was a prominent feature found in cultured hippocampal neurons from offspring of poly I:C-treated mothers. The neuronal M-current encoded by KCNQ2 and KCNQ3 potassium channel subunits was shown to be centrally involved in this property^53^,^54^,^55^. Alternatively, the SK2 small conductance Ca^2+^-activated K^+^ channels, known to be highly expressed in hippocampal pyramidal neurons may also contribute to the enhanced spike frequency adaptation^56^.

Interestingly, a recent study using the poly I:C model of MIA in mice showed an increase in intrinsic excitability of hippocampal dentate gyrus granule cells in 3 months-old offspring from poly I:C-treated mothers^57^. An increased input resistance and a decreased threshold current in both mature and adult-born neurons reflected these alterations. In addition, a substantial reduction of the after-hyperpolarization was found in adult-born granule cell neurons from the poly I:C group^57^. Concomitant with the functional defects, a reduction of the dentate gyrus volume and a decreased number of parvalbumin interneurons were observed following MIA^57^. These data suggest that following MIA different profiles of excitability changes can occur depending on the type of neurons and its location in the hippocampal circuit.

A previous study performed in a mouse model of MIA showed that CA1 pyramidal neurons from hippocampal slices of adult offspring of poly I:C-treated mothers, display reduced frequency and increased amplitude of mEPSCs^58^. However, our data indicate that no changes occurred in mEPSCs characteristics at the neonatal/early postnatal stage. More recently, experiments done in offspring (PND28-31) from poly I:C-injected pregnant rats, showed significantly lower presynaptic fiber volley amplitudes and CA1 pyramidal field excitatory postsynaptic potential slopes^45^. Compared to our study, these differences suggest that the changes in mEPSCs characteristics may occur later in neuronal development.

In our work, the stronger inhibitory GABAergic drive we observed in cultured hippocampal neurons from offspring of the poly I:C group contrasts with the down-regulation of GABAergic pathways usually detected in the hippocampus of rodent model of MIA^17^,^40^,^59^,^60^. A similar increase in GABAergic transmission was previously reported in CA1 pyramidal neurons of organotypic hippocampal slices prepared from neonatal P8–10 rats, where chronic inflammation was produced by a seven days treatment with LPS, leading to significantly lower membrane resistance, lower frequency of action potential discharge and larger evoked IPSCs^42^. This previous study showed that the chronic inflammation induced by LPS was mediated by the release of the inflammatory cytokine IL-1β. Similarly, other works showed that prenatal poly I:C treatment also increased cytokine IL-1β levels in embryonic brains^61^. Another study showed that MIA induced by LPS in rats, dynamically affects offspring spatio-temporal development of pyramidal neurons in medial prefrontal cortex (PND10, 35) and of CA1 hippocampal neurons (PND60), which can potentially lead to aberrant neuronal connectivity and functions in these structures^62^. In our work, the higher charge transfer of sIPSCs found in hippocampal neurons of offspring from poly I:C-injected mothers could be partially accounted for by the larger amplitude of mIPSCs. As no change was found in the frequency of mIPSCs, this feature may be suggestive of larger number of GABA_A_ post-synaptic receptors. A previous study showed that MIA evoked by poly I:C injection of pregnant mice, resulted in adult offspring in a significant increase in GABA_A_ receptor α2 subunit immunoreactivity in the ventral dentate gyrus and basolateral amygdala^63^. Interestingly, a recent work showed that mice prenatal poly I:C treatment produced in the offspring, age-dependent changes in prefrontal cortex expression of GABAergic gene markers^64^. For example, the α2 and α4 subunits of GABA_A_ receptors were up-regulated at young peripubertal age, while their expression levels significantly decreased at the adult stage^64^. The enhanced GABAergic signaling found in our study may reflect early abnormalities of the neonatal stage. Thus, differences in age, specific brain regions and neuronal cell populations may account for distinct GABergic abnormality profiles.

Our data suggest that following MIA, neurophysiological abnormalities occur at the earliest stages of neonatal hippocampal development, possibly via epigenetic alterations. Changes in neuronal intrinsic excitability and other abnormalities occurring at such critical stage of hippocampal development, may potentially lead to subsequent alterations in the maturation of neuronal circuits^65^. Animal modeling provides approaches to ask whether prenatal infection can actually cause transient or lasting changes in brain functions^1^,^2^,^6^,^8^,^9^,^66^. However, the exact mechanisms of how infectious agents lead to abnormal neurodevelopment remain poorly understood^1^,^2^. In addition, most animal models that use prenatal immune activation usually employ high doses of immune activating compounds, beyond what would be physiologically relevant during natural infection^1^,^2^, which should incite us to cautiousness regarding the parallels that are often made between viral infection *in utero* and schizophrenia^1^. Given the genetic component related to schizophrenia risk, it was suggested that experimental studies should focus on studying interactions between prenatal infection and susceptibility genes^1^. Integrating gene-environment interactions in animal model would clearly improve our understanding of MIA as it relates to the pathogenesis of schizophrenia^1^.

## Methods

### Animals

Adult (350–400 g) Wistar rats were housed 3–4 to a cage under reversed cycle lighting (lights on: 19:00–7:00 h) with ad lib food and water. Animal care and all experimental studies were conducted in accordance with “The Guide and Use of Laboratory Animals prepared by the United States National Academy of Sciences” (National Institutes of Health publication number 86–23, revised 1996) and approved by the Institutional Animal Care and Use Committee at Tel-Aviv University, Israel, (animal welfare assurance number A5010-01).

### Prenatal Poly I:C treatment

Prenatal poly I:C treatment was performed as described previously^23^,^24^,^28^. In all experiments, each experimental group consisted of subjects derived from 6 independent litters, with no more than 1–2 rats from the same litter in any of the experimental groups.

### Hippocampal cell culture

Hippocampi were dissected out from PND0-2 male Wistar rats and cultures were performed as previously^47^. For electrophysiological recordings, hippocampal neurons with pyramidal-like morphology were used at 14–17 days in culture.

### Electrophysiology of cultured hippocampal neurons

Voltage- and current-clamp recordings were performed using the whole-cell configuration of the patch-clamp technique. Data were acquired using pClamp 9.2 software in conjunction with a DigiData 1322A interface (Molecular Devices). The patch pipettes were pulled from borosilicate glass (Warner Instrument) with a resistance of 4–7 MΩ. Series resistances (5–15 MΩ) were compensated (75–90%) and periodically monitored. Spontaneous, miniature excitatory and inhibitory postsynaptic currents (sEPSCs, sIPSCs, mEPSCs and mIPSCs, respectively) were recorded as previously described^67^. Briefly, sEPSCs were recorded at a holding potential of −70 mV. The extracellular solution consisted (in mM) of 160 NaCl, 2.5 KCl, 10 HEPES, 10 glucose, 2 CaCl_2_; pH 7.3 (325 mOsm), to which 30 μM picrotoxin and 10 μM bicuculline methyl iodide were added. The intracellular solution consisted (in mM) of 130 K-gluconate, 10 KCl, 1.1 EGTA, 10 HEPES, 1 MgCl_2_, 2 Na_2_ ATP, 0.1 CaCl_2_ and 5 QX314Br to block Na^+^ currents; pH 7.2 (315 mOsm). sIPSCs were recorded at a holding potential of −70 mV; the extracellular solution contained (in mM) 160 NaCl, 2.5 KCl, 10 HEPES, 10 glucose, 2 CaCl_2_; pH 7.3 (325 mOsm), to which 10 μM 1,2,3,4-tetrahydro-6-nitro-2,3-dioxo-benzo[f]quinoxaline-7-sulfonamide (NBQX) and 10 μM AP-5 were added. The intracellular solution consisted (in mM) of 144 CsCl, 10 HEPES, 1.1 EGTA, 0.1 CaCl_2_, 5 MgCl_2_, 2 Na_2_ATP, 5 QX314; pH 7.3 (315 mOsm). For isolating mEPSC and mIPSC, 1 μM tetrodotoxin was added to the extracellular solution consisting of (in mM) 160 NaCl, 2.5 KCl, 10 HEPES, 10 glucose, 2 CaCl_2_; pH 7.3 (325 mOsm), to which 30 μM picrotoxin and 10 μM bicuculline methyl iodide were added for mEPSC recordings or 10 μM NBQX and 10 μM AP-5 for mIPSC recordings. For current-clamp recordings, the patch pipettes were filled with (in mM): 135 KCl, 1 K_2_ATP, 1 MgATP, 2 EGTA, 1.1 CaCl_2_, 5 glucose, 10 HEPES ; pH 7.4 (315 mOsm). The external solution contains (in mM): 160 NaCl, 2.5 KCl, 2 CaCl_2_, 2 MgCl_2_, 10 glucose, 10 HEPES ; pH7.4 (325 mOsm). For blocking the spontaneous synaptic activity, the above external solution contained 10 μM NBQX, 10 μM AP-5, 30 μM picrotoxin and 10 μM bicuculline methyl iodide. For evoking spike discharges, squared current pulses of −300 pA up to 250 pA were injected into neurons for 800 ms duration. For evoking solitary spike, 200–800 pA depolarizing squared current pulses were injected into neurons for 2 ms duration. All signals were amplified using an Axopatch 200A patch-clamp amplifier (Molecular Devices), sampled at 5 kHz and filtered via a 4-pole Bessel low pass filter, at 1 kHz for mEPSCs and mIPSCs recordings and at 2 kHz for the other recordings. The membrane potential values were corrected for the liquid junction potential (5.0 mV).

### Hippocampal slice preparation

Brains were rapidly removed from male offspring at PNDs 14–16. Coronal hippocampal slices (400 μm thick) were prepared in a cold (4 °C) storage buffer containing (in mM): sucrose, 206; KCl, 2; MgSO4, 2; NaH2PO4, 1.25; NaHCO3, 26; CaCl2, 1; MgCl2, 1; glucose, 10 using a Leica VT1200 vibratome. Slices were immediately transferred to a submerged recovery chamber at room temperature artificial cerebrospinal fluid (aCSF) bubbled with 95% O2 and 5% CO2 for at least 1 h before recording. The aCSF contained (in mM): NaCl, 125; KCl, 2.5; CaCl2, 1.2; MgCl2, 1.2; NaHCO3, 25; NaH2PO4, 1.25; glucose, 25. Compared groups had always the same time of recovery in aCSF.

### Whole cell recording in hippocampal slices

Slices were placed into the recording bath at room temperature and perfused with aCSF bubbled with 95% O_2_ and 5% CO_2_. Single hippocampal CA1 neurons were visualized using infrared optics and recorded with patch pipettes (5–7 MΩ) containing the following (in mM): 120 K-gluconate, 10 KCl, 10 HEPES, 10 Na phosphocreatine, 0.5 EGTA, 4 Na_2_-ATP, 0.4 GTP, 0.5 MgCl_2_ adjusted to pH 7.2 with KOH. Data were acquired using pClamp 10.3 software (Molecular Devices) in conjunction with a multiclamp700B interface digitized with DigiData 1440A (Molecular Devices). For input resistance measurements negative current step of −150 pA was injected into the cell. For evoking spike discharges, squared current pulses of 0 pA up to 225 pA in 25 pA increments were injected into neurons during 400 ms. Solitary spikes (for rheobase current measurements) were evoked by 2 ms depolarizing squared current pulses from 50 pA in 50 pA increments. Current clamp recordings were sampled at 10 kHz and filtered via a 4-pole Bessel low pass filter, at 5 kHz. The membrane potential values were corrected for the liquid junction potential (14.8 mV).

### Data analysis

Data analysis was performed using the Clampfit program (pClamp 9.2; Molecular Devices), Microsoft Excel (Microsoft) and Prism 4.0 (GraphPad). All data were expressed as means ± SEM. Statistically significant differences were assessed by unpaired Student’s t-test (two-tailed) with a significance level of p < 0.05, except where mentioned. For statistical significance of evoked spiking activity one-way ANOVA with main factor of prenatal treatment with repeated-measure factor of current injection was performed. Significant interactions were followed by least significant difference post hoc comparisons. Analysis of mEPSCs and mIPSCs included evaluation of the individual events (for each recorded neuron, 200 events were analyzed from mEPSC recordings and 50 events from mIPSC recordings). Event detection was performed using the mini analysis program (Synaptosoft). Statistical evaluation of the cumulative probability of amplitude and frequency (inter-event intervals) was performed with the Kolmogorov-Smirnov nonparametric two-sample test with a significance level of P < 0.001. For the mean decay time and rise time, statistical evaluation was assessed by unpaired Student’s t-test (two-tailed).

## Additional Information

**How to cite this article**: Patrich, E. *et al.* Maternal immune activation produces neonatal excitability defects in offspring hippocampal neurons from pregnant rats treated with poly I:C. *Sci. Rep.* **6**, 19106; doi: 10.1038/srep19106 (2016).

## Figures and Tables

**Table 1:** Intrinsic excitability parameters of cultured hippocampal neurons from offspring of saline and polyI:C-injected moms.

	Resting potential (mV)	Input resistance^a^ (MΩ)	Threshold potential^b^ (mV)	AP amplitude (mV)	AP width at threshold (ms)	Rheobase^c^ (pA)
Saline-injected mothers	−59.3 ± 1.2 (n = 44)	236 ± 8.8 (n = 41)	−33.6 ± 0.6 (n = 37)	81.2 ± 1.7 (n = 44)	2.40 ± 0.15 (n = 44)	409 ± 19 (n = 44)
PolyI:C injected mothers	−60.5 ± 1.0 (n = 54)	200.5 ± 8.5 ** (n = 56)	−34.5 ± 0.6 (n = 55)	87.2 ± 1.7 * (n = 54)	2.10 ± 0.08 * (n = 54)	594 ± 25 ** (n = 54)

The parameters were measured in numbers of neurons (n) from offspring taken from 6 different mothers (N). The membrane potential values were corrected for the liquid junction potential (5.0 mV).

* and ** indicates statistical significance of p < 0.05 and p < 0.01, respectively (assessed by two-sample assuming equal variances t-test).

^a^Calculated from the voltage change following a −300 pA current injection for 800 ms (Rin = V/I in MΩ).

^b^Measured as the potential at the beginning of the AP upstroke.

^c^The rheobase current was determined by injecting neurons with a series of current steps starting from 200 pA, followed by 50 pA increments steps for a 2 ms pulse duration, until a single action potential (AP) was elicited.
